# Supplementary material for: A high-gain cladded waveguide amplifier on erbium doped thin-film lithium niobate fabricated using photolithography assisted chemo-mechanical etching
Source: Nanophotonics. 2022 Jan 17;11(5):1033–40. doi: 10.1515/nanoph-2021-0737 (PMC11501891; doi:10.1515/nanoph-2021-0737)
Supplement: Supplementary file 1 — Supplementary Material [file j_nanoph-2021-0737_suppl.docx]

**Supplemental materials for**

**A high-gain cladded waveguide amplifier on erbium doped thin-film lithium niobate fabricated using photolithography assisted chemo-mechanical etching**

Youting Liang^1, 2^, Junxia Zhou^1, 2^, Zhaoxiang Liu^2^, Haisu Zhang^1, 2, *^, Zhiwei Fang^2^, Yuan Zhou^3^, Difeng Yin^3^, Jintian Lin^3^, Jianping Yu^3^, Rongbo Wu^3^, Min Wang^2^, and Ya Cheng^1, 2, 3, 4, 5, 6, *^

^1^State Key Laboratory of Precision Spectroscopy, East China Normal University, Shanghai 200062, China.

^2^The Extreme Optoelectromechanics Laboratory (XXL), School of Physics and Electronic Sciences, East China Normal University, Shanghai 200241, China.

^3^State Key Laboratory of High Field Laser Physics and CAS Center for Excellence in Ultra-Intense Laser Science, Shanghai Institute of Optics and Fine Mechanics (SIOM), Chinese Academy of Sciences (CAS), Shanghai 201800, China.

^4^Shanghai Research Center for Quantum Sciences, Shanghai 201315, China.

^5^Collaborative Innovation Center of Extreme Optics, Shanxi University, Taiyuan 030006, China. ^6^Collaborative Innovation Center of Light Manipulations and Applications, Shandong Normal University, Jinan 250358, China.

*Correspondence: Haisu Zhang (hszhang@phy.ecnu.edu.cn), Ya Cheng (ya.cheng@siom.ac.cn).

In the Supplemental materials we describe the waveguide fabrication process by PLACE and the theoretical model for the erbium-doped waveguide amplifier.

## The waveguide fabrication process by PLACE


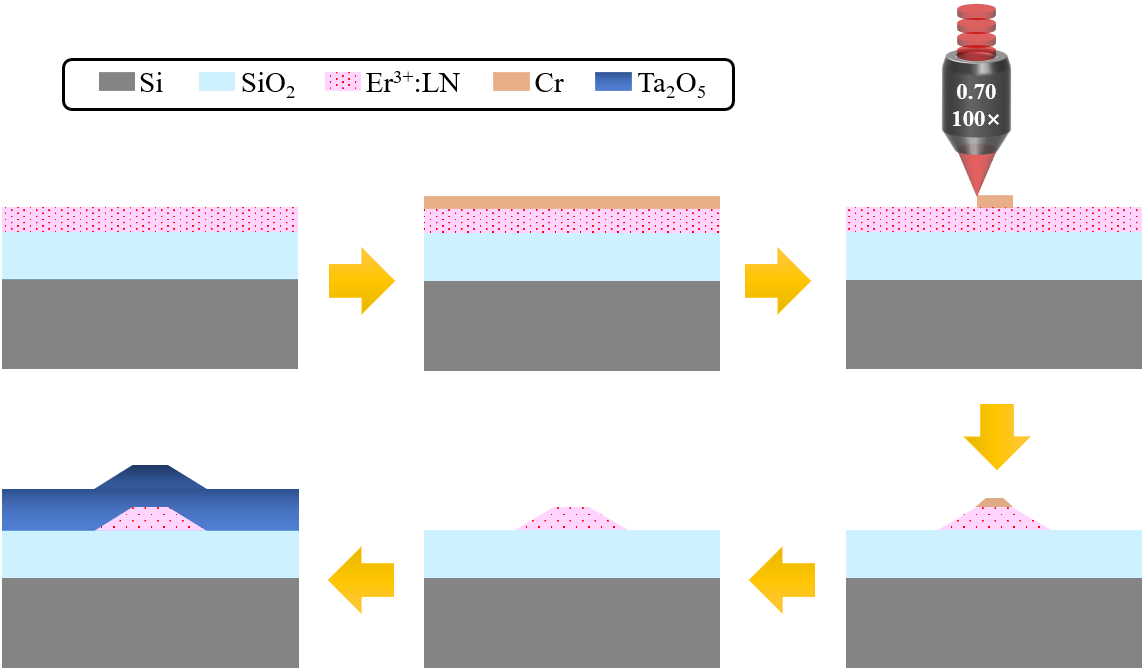


Fig. S1 Schematic illustration of waveguide fabrication process by PLACE.

The waveguide fabrication procedures by PLACE are schematically illustrated in Fig. S1, including (1) deposition of a thin layer of chromium (Cr) with a thickness of 400 nm on the LNOI wafer by magnetron sputtering, and (2) patterning of the Cr film using femtosecond laser ablation, (3) pattern transferring from the Cr mask to the underneath LN thin film by chemo-mechanical polishing (CMP), (4) removal of remaining Cr mask and a secondary CMP. After the waveguide is shaped, a thin layer of Ta_2_O_5_ is deposited on top of LNOI by sputtering coating. The smooth sidewalls of the fabricated LNOI waveguides are guaranteed by CMP which can maintain subnanometer surface/interface roughness comparable with the surface-tension induced atomic scale finish.

## The theoretical model of Er^3+^-doped waveguide amplifier

The Er^3+^-doped amplifier model introduced in Refs. [8, 9] is employed, which includes the steady-state response of the three-level Er^3+^ with ^4^I_15/2_, ^4^I_13/2_, and ^4^I_11/2_, and the migration-accelerated ETU process and the concentration quenching of erbium ions. The involved transitions are shown in Fig. S2.


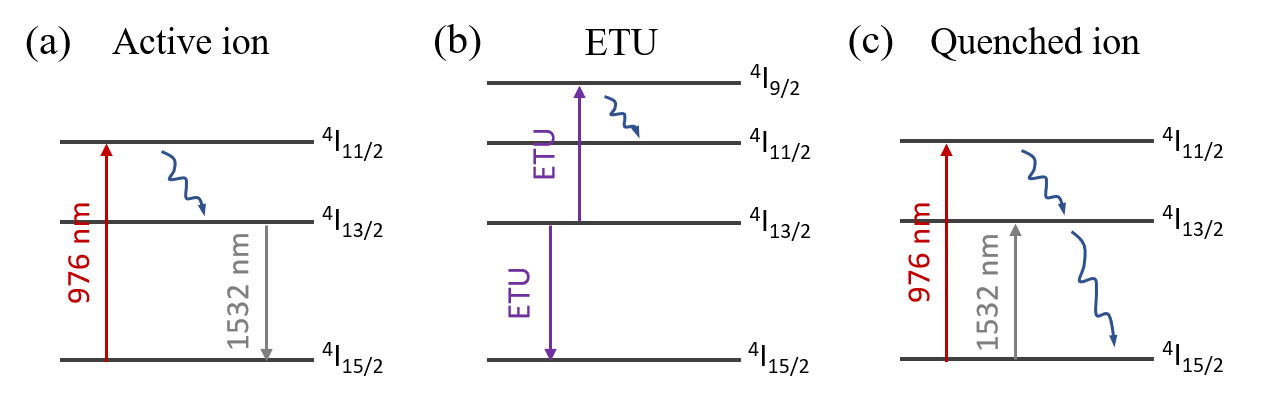


Fig. S2 The involved Er^3+^ energy levels in the amplifier model. (a) the 3-level system for active ions. (b) the migration-accelerated ETU process. (c) the 3-level system for quenched ions. The blue wavy arrows denote fast decay.

The steady-response of Er^3+^ in the pump and signal fields can be obtained by the equations below:

$\frac{dN_{2}}{dt}=F_{P}\left( \sigma_{a}^{P}N_{0}-\sigma_{e}^{P}N_{2} \right)+W_{ETU}N_{1}^{2}-\frac{1}{\tau_{2}}N_{2}=0$ (1)

$\frac{dN_{1}}{dt}=F_{s}\left( \sigma_{a}^{S}N_{0}-\sigma_{e}^{S}N_{1} \right)+\frac{1}{\tau_{2}}N_{2}-2W_{ETU}N_{1}^{2}-\frac{1}{\tau_{1}}N_{1}=0$ (2)

$N_{0}+N_{1}+N_{2}=N_{d}$ (3)

where $N_{i}$(i=0, 1, 2) denote the population density of ^4^I_15/2_, ^4^I_13/2_, and ^4^I_11/2_ states, $N_{d}$ is the total density of Er^3+^, $\tau_{2}$ is the non-radiative decay time from ^4^I_11/2_ to ^4^I_13/2_, $\tau_{1}$ is the intrinsic decay time of ^4^I_13/2_, $W_{ETU}$ is the macroscopic migration parameter for (^4^I_13/2_, ^4^I_13/2_)→(^4^I_15/2_, ^4^I_9/2_), $\sigma_{a}^{P}$($\sigma_{a}^{S}$) and $\sigma_{e}^{P}$($\sigma_{e}^{S}$) are the absorption and emission cross sections for the pump (signal) light. $F_{P}={I_{P}}/{h\nu_{P}}$ and $F_{P}={I_{S}}/{h\nu_{S}}$ are the photon fluxes of the pump and signal light, with $I_{P}$($I_{P}$) and $\nu_{P}$($\nu_{S}$) being the pump (signal) intensity and frequency. The excited population of the ^4^I_9/2_ level by ETU is not included since it will decay to the ^4^I_11/2_ level by fast multiphonon relaxation in less than 1 μs.

For the concentration quenching, a small fraction ($f_{q}$) of Er^3+^ is assumed to decay rapidly from excited states by static ETU among active ion-pairs or clusters, and excited state trapping by host material defects, resulting in a luminescence lifetime of quenched ions on the order of 1 μs. The response of quenched ions can also be described by equations (1)-(3) with the replacement of the total ion density $N_{d}$ and the excited level lifetimes $\tau_{2}$&$\tau_{1}$ by ${f_{q}N}_{d}$ and $\tau_{q}$, respectively. The total optical gain is obtained by summing up the steady-state responses of active ions${(1-f_{q})N}_{d}$ and quenched ions ${f_{q}N}_{d}$.

Excited-state absorption (ESA) at the pump wavelength is included as a loss factor in the light propagation along the Er^3+^-doped waveguide, which written as:

$\frac{dP_{P}\left( z \right)}{dz}=P_{P}\left( z \right)\left[ \iint\Psi_{P}\left( x,y \right)\left( \sigma_{e}^{P}N_{2}-\sigma_{ESA}^{P}N_{2}-\sigma_{a}^{P}N_{0} \right)dxdy-\alpha\right]$ (4)

$\frac{dP_{S}\left( z \right)}{dz}=P_{S}\left( z \right)\left[ \iint\Psi_{S}\left( x,y \right)\left( \sigma_{e}^{S}N_{1}-\sigma_{a}^{S}N_{0} \right)dxdy-\alpha\right]$ (5)

$P_{P}\left( z \right)$ and $P_{S}\left( z \right)$ are the pump and signal powers at the waveguide length $z$, $\alpha$ is the waveguide passive propagation loss. $\Psi_{P}\left( x,y \right)$ and $\Psi_{S}\left( x,y \right)$ are the normalized modal field intensities at the pump and signal wavelengths obtained from finite element simulation.

The utilized parameters for Er^3+^:LNOI waveguide amplifier are listed in table S1.

| Parameter | Value | Source |
| --- | --- | --- |
| $\sigma_{a}^{S}$ | 1.95×10^-20^ cm^2^ | [27] |
| $\sigma_{e}^{S}$ | 1.55×10^-20^ cm^2^ | [27] |
| $\sigma_{a}^{P}$ | 0.85×10^-20^ cm^2^ | [27] |
| $\sigma_{e}^{P}$ | 0 | [27] |
| $\sigma_{ESA}^{P}$ | 1.0×10^-22^ cm^2^ | [28] |
| $W_{ETU}$ | 1.0×10^-18^ cm^3^/s | [28] |
| $\tau_{2}$ | 200 μs | [28] |
| $\tau_{1}$ | 2.35 ms | [29] |
| $\tau_{q}$ | 1 μs | [9] |
| $f_{q}$ | 0, 0.1, 0.2 |  |
| $\lambda_{P}$ | 976 nm |  |
| $\lambda_{S}$ | 1532 nm |  |
| $\alpha$ | 0.1 dB/cm |  |
| $N_{d}$ | 1.9×10^20^ cm^-3^ |  |
